# Prevalence of Sarcopenia in Patients with Hip Osteoarthritis Undergoing Total Hip Arthroplasty: A Cross-Sectional Study Based on EWGSOP2 Criteria

**DOI:** 10.3390/jcm15156050

**Published:** 2026-08-04

**Authors:** Marcos Del Carmen-Rodríguez, Blanca Plana-Villa, Daniel Rodríguez-Pérez, Carles Tramunt-Montsonet, Carmen Gómez-Vaquero, Jose Luis Agulló-Ferré

**Affiliations:** 1 Faculty of Medicine and Health Sciences, Universitat de Barcelona (UB), C. Casanova, 143, 08036 Barcelona, Spain; carmen.gomez@bellvitgehospital.cat (C.G.-V.); jlagullo@bellvitgehospital.cat (J.L.A.-F.); 2 Orthopaedic Surgery and Traumatology Department, Hospital Universitari de Bellvitge, L’Hospitalet de Llobregat, 08907 Barcelona, Spain; bplanavilla@gmail.com (B.P.-V.); d.rodriguez.perez@bellvitgehospital.cat (D.R.-P.); ctramunt@bellvitgehospital.cat (C.T.-M.); 3 Rheumatology Department, Hospital Universitari de Bellvitge, L’Hospitalet de Llobregat, 08907 Barcelona, Spain

**Keywords:** Sarcopenia, EWGSOP2, total hip arthroplasty, DXA, hip osteoarthritis

## Abstract

(1) **Background/Objectives**: Sarcopenia is common in older surgical candidates and may affect perioperative risk and recovery after total hip arthroplasty (THA). Quantifying its burden and severity gradient in THA pathways can inform surgeon-led optimization. (2) **Methods**: A cross-sectional analysis of baseline preoperative data at a tertiary center (December 2019 to December 2022) was conducted. Consecutive adults with hip osteoarthritis undergoing primary THA were screened. Sarcopenia was staged per EWGSOP2: probable (low handgrip), confirmed (probable and low skeletal muscle index by whole-body DXA), and severe (confirmed and low gait speed). (3) **Results**: Of 312 screened patients, 203 completed baseline assessment (95 men, 108 women). According to the EWGSOP2 algorithm, 57 patients (28.1%; 95% CI 22.3–34.6%) had low handgrip strength, 30 (14.8%; 95% CI 10.6–20.3%) fulfilled criteria for confirmed sarcopenia, and 21 (10.3%; 95% CI 6.9–15.3%) had severe sarcopenia. No significant association was observed for sex. Sarcopenia diagnosis was associated with older age, lower body mass index (BMI), higher comorbidity burden, lower functional independence, greater cognitive impairment, poorer mental health, and worse hip-related function (all *p* < 0.05). In multivariable analysis, older age (OR 1.51 per 5-year increase; 95% CI 1.11–2.12), lower BMI (OR 0.88 per kg/m^2^; 95% CI 0.79–0.98), and lower functional independence (Barthel Index: OR 2.02 per 10-point decrease; 95% CI 1.20–3.60) were independently associated with confirmed sarcopenia. Across EWGSOP2 categories, differences in age, BMI, pain, comorbidity, functional independence, cognition, mental health and hip function showed significant differences (all *p* < 0.05), with ordered trends in most domains but no uniform monotonic pattern. (4) **Conclusions**: In THA candidates, sarcopenia is prevalent and is related to older age, lower BMI and lower functional independence. Integrating sarcopenia screening by EWGSOP2 may help to identify THA candidates with greater clinical vulnerability who may warrant further preoperative assessment and targeted optimization strategies.

## 1. Introduction

Sarcopenia is an age-related, progressive skeletal muscle disorder characterized by the loss of skeletal muscle mass and function [1]. In 2010, the European Working Group on Sarcopenia in Older People (EWGSOP) established unified diagnostic criteria to facilitate the identification and treatment of Sarcopenia worldwide [2]. EWGSOP2 updated this consensus in 2018, incorporating the latest scientific evidence accumulated over the previous decade. This revision emphasized low muscle strength as the primary parameter for the diagnosis of sarcopenia in clinical practice and defined sarcopenia as severe when physical performance is impaired [3]. Sarcopenia has been linked to an increase in falls, fractures, disability, and mortality, with potential effects on postoperative outcomes, which demonstrates its perioperative relevance [3,4,5].

Osteoarthritis (OA) is recognized as a contributor to secondary sarcopenia, as chronic pain, reduced mobility, and low-grade systemic inflammation associated with the disease promote physical inactivity and muscle catabolism [6]. Moreover, emerging evidence suggests that reductions in lean mass may increase the risk of developing hip OA through biomechanical instability and systemic health impairment [6,7]. In this context, sarcopenia and OA appear as closely interrelated conditions: OA-related pain and functional limitation promote muscle loss, while declining muscle mass and strength may further compromise joint stability and accelerate disease progression [8].

Sarcopenia—or the risk of developing it—is common among patients undergoing arthroplasty. Using SARC-F, a validated questionnaire for identifying patients at risk of sarcopenia, 72.3% of adults scheduled for total hip arthroplasty (THA) screened positive [9]. Beyond prevalence, sarcopenia has significant implications for outcomes after THA in patients with OA, including increased risk of dislocation and aseptic loosening, delayed functional recovery, poorer patient-reported outcomes (PROs), higher incidence of urinary tract infections, greater 90-day readmissions, elevated risk of falls and fragility fractures—particularly in older adults—and increased episode-of-care costs [5,10,11,12]. A systematic review across hip and knee arthroplasty studies corroborates the adverse impact of sarcopenia on function and independence [13].

Consequently, the quantitative assessment of sarcopenia and the analysis of its severity represent a significant clinical strategy for orthopedic surgeons. Based on this reasoning, the aim of this study is to quantify the prevalence and severity of sarcopenia in a consecutive cohort of patients undergoing THA and to assess the association with a range of clinical variables.

## 2. Materials and Methods

This study reports a cross-sectional analysis of baseline data from a single-center prospective observational cohort of consecutive adults aged 50–90 years with unilateral or bilateral hip OA undergoing THA between 2019 and 2022 at Hospital Universitari de Bellvitge (L’Hospitalet de Llobregat, Barcelona, Spain). Exclusion criteria were conditions associated with secondary OA (proximal femoral deformity, femoral head osteonecrosis, inflammatory disease), conditions associated with sarcopenia (severe chronic and inflammatory diseases), major psychiatric disorders, severe lower-limb articular pathology, and BMI > 40 kg/m^2^. These criteria were applied to minimize clinical heterogeneity, thereby improving the internal validity for the evaluation of sarcopenia in the cohort.

The study protocol was approved by the local ethics committee (PR217/19, 21 November 2019). All included participants provided written informed consent.

### 2.1. Sarcopenia Diagnostic Variables

Sarcopenia was diagnosed according to the EWGSOP2 criteria [3]. Muscle strength was assessed by handgrip strength, measured using a hydraulic dynamometer (Saehan SH5001, Saehan Corporation, Yangsan, Republic of Korea). Measurements were performed in the dominant hand with the handle set in the second position. Participants were seated with the elbow flexed at 90°, and three maximal contractions were recorded; the mean value was used for analysis. Probable sarcopenia was defined as handgrip strength <27 kg in men and <16 kg in women. Sarcopenia was confirmed by low muscle quantity assessed using whole-body composition dual-energy X-ray absorptiometry (DXA) (Horizon Wi, Hologic Inc., Bedford, MA, USA). Scans were performed with the participants supine, centered on the scanning table, with the arms alongside the body and hands facing the thighs without contact. Appendicular lean mass was used to compute the skeletal muscle index (SMI = appendicular lean mass/height^2^), and sarcopenia was confirmed when SMI < 7.0 kg/m^2^ in men or < 5.5 kg/m^2^ in women. To assess sarcopenia severity, gait speed was measured using the 6-m gait speed test. Participants walked at their usual pace and began walking before entering the timed 6-m course, allowing a flying start. A single trial time was recorded using a stopwatch, without walking aids or physical assistance, and gait speed was calculated in meters per second (m/s). Severe sarcopenia was defined as gait speed ≤ 0.8 m/s.

**Figure 1:**
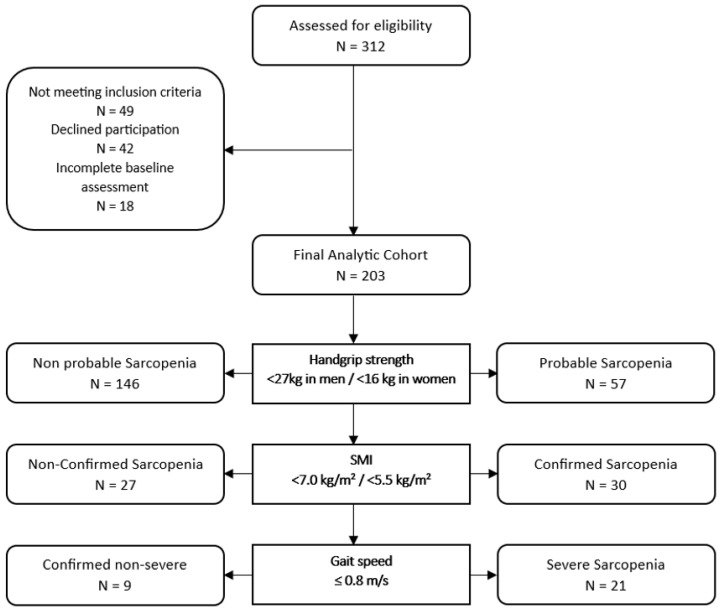
Study flow diagram and EWGSOP2 staging. Flow of participants from eligibility assessment to the final analytic cohort, and stepwise EWGSOP2 classification using handgrip strength, SMI, and gait speed for severe sarcopenia. Cut-offs according to EWGSOP2; gait speed measured over 6 m; SMI derived from DXA. Reasons for ineligibility (*n* = 49) included secondary hip osteoarthritis or proximal femoral deformity (*n* = 12), femoral head osteonecrosis (*n* = 9), inflammatory disease (*n* = 6), severe chronic disease or oncological disease associated with sarcopenia (*n* = 7), major psychiatric disorders (*n* = 3), severe lower-limb joint articular pathology (*n* = 4), and BMI > 40 kg/m^2^ (*n* = 8).

Participant selection and EWGSOP2-based classification are summarized in Figure 1.

### 2.2. Demographic and Anthropometric Characteristics

This domain included age, sex, and body mass index (BMI). Age was categorized into four groups (50–59, 60–69, 70–79, and ≥80 years). Sex was classified into male or female. BMI (kg/m^2^) was classified as underweight (<18.5), normal weight (18.5–24.9), overweight (25–29.9), class I obesity (30–34.9), class II obesity (35–39.9), and class III obesity (≥40).

### 2.3. Baseline Clinical Status and Health-Related Measures

Baseline clinical status and health-related measures were assessed using a combination of clinical, laboratory, functional, cognitive, and PRO variables. Pain was measured using the Visual Analog Scale (VAS).

Laboratory parameters included serum hemoglobin and serum albumin concentrations.

Patients’ preoperative physical status was classified according to the American Society of Anesthesiologists (ASA) Physical Status Classification [14]. Preoperative physical status was classified from I to IV: I, healthy patient; II, patient with mild systemic disease; III, patient with severe systemic disease; IV, patient with severe systemic disease that is a constant threat to life. The score was assigned by the attending anesthesiologist during the preoperative assessment.

Comorbidity burden was evaluated using the original Charlson Comorbidity Index, which assigns weighted points to predefined comorbid conditions to generate a summary score, with higher values indicating greater comorbidity burden [15].

Functional independence was quantified with the Barthel Index [16] (range 0–100, with higher scores indicating greater independence in activities of daily living).

Cognitive function was evaluated using the Global Deterioration Scale (GDS) described by Barry Reisberg (stages 1–7), with lower scores reflecting better cognitive status: 1 indicates no cognitive decline, 2–3 mild cognitive impairment, 4 moderate cognitive decline, 5 moderately severe decline, 6 severe decline, and 7 very severe cognitive decline [17].

Health-related quality of life was measured with the 12-Item Short Form Health Survey (SF-12, version 1), including the Physical Component Summary (PCS) and Mental Component Summary (MCS) scores, where higher values indicate better perceived physical and mental health [18].

### 2.4. Radiographic Characteristics of the Proximal Femur

Radiological evaluation included the assessment of radiographic OA using the Tönnis grading scale [19] (grades 0–3, where 0 indicates no OA and 3 severe OA), as well as the characterization of proximal femur morphology using the Dorr classification (types A-C) [20], the Canal-to-Calcar Ratio (CCR) [21], and the Cortical Thickness Index (CTI) [22]. In the Dorr classification, type A indicates a narrow canal with thick cortices, type B an intermediate morphology, and type C a wide canal with thin cortices. The CCR was calculated as the ratio of the femoral canal width measured 100 mm distal to the lesser trochanter to the calcar canal width. Higher values indicate a wider canal and thinner cortices. The CTI was calculated as the ratio of the combined medial and lateral cortical thickness to the total femoral width at 100 mm distal to the lesser trochanter. This index reflects the relative cortical thickness of the femoral diaphysis on anteroposterior radiographs, with lower values indicating thinner cortices.

### 2.5. Functional Outcomes

Functional outcomes were assessed using validated functional and PROs, including the modified Harris Hip Score (mHHS) [23], which assesses pain and hip function with higher scores indicating better function; the Western Ontario and McMaster Universities Osteoarthritis Index (WOMAC) [24], which evaluates pain, stiffness, and physical function, with higher scores indicating worse symptoms and functional limitation; and the HOOS-12 (12-item Hip disability and Osteoarthritis Outcome Score) [25], a short-form PRO measure assessing hip-related pain, function, and quality of life, with higher scores reflecting better hip-related outcomes.

### 2.6. Statistical Analysis

The study was designed in 2019, and the sample size was estimated a priori for a cross-sectional prevalence study based on the expected prevalence of sarcopenia and a predefined absolute precision. Based on previously reported prevalence rates ranging from 10.5% to 28.9% in patients undergoing THA [26], with a 95% confidence level and an absolute precision of approximately 6.5%, the required sample size was estimated to be 187 patients using the upper prevalence estimate. Allowing for potential missing data or incomplete assessments, a target sample size of approximately 200 patients was considered adequate and feasible for estimating sarcopenia prevalence in this clinical cohort. Quantitative variables were summarized as mean ± SD or median [IQR], as appropriate, and categorical variables as counts and percentages (*n*, %). Normality was assessed using the Shapiro-Wilk test.

Variables that form part of the EWGSOP2 case definition (handgrip strength, gait speed, and SMI) were not inferentially compared across sarcopenia categories to avoid incorporation bias. However, sex-stratified descriptive comparisons were reported to characterize baseline differences in EWGSOP2 definitional measures.

The primary analysis estimated the prevalence of probable, confirmed, and severe sarcopenia according to EWGSOP2 criteria, as well as the distribution of participants across the four mutually exclusive EWGSOP2 categories. Prevalence estimates were reported with 95% confidence intervals calculated using the Wilson score method.

The first exploratory comparative analysis examined baseline characteristics in patients with and without confirmed sarcopenia. Categorical variables were compared using the χ^2^ test or Fisher’s exact test when appropriate, and continuous variables using the Student’s t-test/ANOVA or Mann–Whitney U test as appropriate. To account for multiple testing, *p*-values from these comparisons were adjusted using the Benjamini–Hochberg false discovery rate (FDR) procedure. Adjusted *p*-values were reported. Factors independently associated with confirmed sarcopenia were examined using a prespecified Firth penalized logistic regression model including age, sex, BMI, and Barthel Index, entered simultaneously without automated variable selection. Age was modeled per 5-year increase and Barthel Index per 10-point decrease, and adjusted odds ratios (ORs) with 95% confidence intervals (CIs) were reported. Multicollinearity and linearity in the logit were assessed, and a conventional enter-method logistic regression including the same covariates was performed as a sensitivity analysis. Model calibration and discrimination were evaluated using the Hosmer-Lemeshow test and the area under the receiver operating characteristic curve, respectively.

A secondary exploratory comparative analysis examined overall differences and ordered trends across four mutually exclusive EWGSOP2 categories (0 = No sarcopenia, 1 = Probable sarcopenia only, 2 = Confirmed non-severe sarcopenia, 3 = Severe sarcopenia). Overall between-group differences were assessed using the Kruskal–Wallis test for continuous and ordinal variables and the Fisher–Freeman–Halton exact test with Monte Carlo estimation for categorical variables. Because these categories reflect a priori sequential accumulation of EWGSOP2 diagnostic criteria, the Jonckheere–Terpstra test was additionally used as a complementary ordered-trend analysis for continuous and ordinal outcomes. This test assessed an overall ordered distributional shift and did not assume a linear relationship, equal spacing between categories, or strictly monotonic group medians. Ordered-trend analyses were not applied to nominal categorical variables. *p*-values from the overall between-group and ordered-trend analyses were adjusted separately using the Benjamini–Hochberg FDR procedure. All tests were two-sided and were performed on available cases (no imputation).

All statistical analyses were conducted using IBM SPSS Statistics v25 (IBM Corp., Armonk, NY, USA).

## 3. Results

A total of 203 patients were included in the final analytic cohort (Figure 1): 95 men (46.8%) and 108 women (53.2%). Mean age was 69.3 ± 8.5 years, with men being younger than women (67.8 ± 8.4 vs. 70.7 ± 8.4 years; *p* = 0.015). EWGSOP2 definitional measures differed by sex: handgrip strength was higher in men than in women (36.0 [28.8–40.25] kg vs. 18.95 [15.7–22.45] kg; *p* < 0.001), SMI was higher in men than in women (7.06 ± 0.76 vs. 5.73 ± 0.84 kg/m^2^; *p* < 0.001), and 6-m walking speed was higher in men than in women (0.93 ± 0.28 vs. 0.78 ± 0.24 m/s; *p* < 0.001).

According to the EWGSOP2 diagnostic algorithm, 57 patients (28.1%; 95% CI 22.3–34.6%) met the criteria for probable sarcopenia based on low handgrip strength. Of these, 30 patients (14.8%; 95% CI 10.6–20.3%) also had low SMI and were therefore classified as having confirmed sarcopenia; among them, 21 patients (10.3%; 95% CI 6.9–15.3%) additionally showed low physical performance and fulfilled criteria for severe sarcopenia.

Sex-stratified analyses were not performed, as neither confirmed nor severe sarcopenia differed by sex.

Patients with confirmed sarcopenia were significantly older (*p* < 0.01), had lower BMI (*p* < 0.05), higher comorbidity burden as measured by the original Charlson Comorbidity Index (*p* < 0.05), lower functional independence (Barthel Index, *p* < 0.01), greater cognitive impairment (GDS, *p* < 0.001), poorer mental health status (SF-12 MCS, (*p* < 0.05) and worse hip-related function (mHHS, *p* < 0.05). No significant differences were found for the remaining variables (Table 1).

Across EWGSOP2 severity categories (Table 2), overall between-group analyses showed significant differences after FDR adjustment for age (*p* < 0.001), pain intensity (VAS, *p* = 0.013), comorbidity burden (original Charlson Comorbidity Index, *p* < 0.001), functional independence (Barthel Index, *p* < 0.001), cognitive status (GDS, *p* < 0.001), and mental health (SF-12 MCS, *p* < 0.001). Regarding PROs, significant differences after FDR adjustment for hip function (mHHS, *p* < 0.001), HOOS-12 Quality of Life (*p* < 0.05), and WOMAC total, Symptoms, and Pain scores (all *p* < 0.05). Complementary ordered-trend analyses supported an overall ordered association for age, pain intensity, comorbidity burden, functional independence, cognitive status, mental health, hip function (all *p* < 0.003), HOOS-12 Quality of Life (*p* = 0.009), WOMAC total (*p* = 0.034), and WOMAC Symptoms (*p* = 0.005). However, these patterns were not uniformly monotonic across all four categories. In the sensitivity analysis combining confirmed non-severe and severe sarcopenia, the principal between-group differences and ordered associations were preserved, indicating that the main findings were not solely dependent on the gait-speed-based severity classification. No significant overall differences were observed for the rest of the variables.

Multivariable analyses were performed to examine variables independently associated with sarcopenia diagnosis. Variables showing significant between-group differences in univariable analyses, together with age and sex as clinically relevant covariates (BMI and Barthel Index), were considered for model selection. The overall model was statistically significant (*p* < 0.001). Older age was independently associated with confirmed sarcopenia (OR 1.51 per 5-year increase; 95% CI 1.11–2.12; *p* = 0.008). Each 10-point decrease in the Barthel Index was associated with approximately twofold higher odds of confirmed sarcopenia (OR 2.02; 95% CI 1.20–3.60; *p* = 0.008), whereas higher BMI was inversely associated with sarcopenia (OR 0.88 per kg/m^2^; 95% CI 0.79–0.98; *p* = 0.018). Sex was not independently associated with confirmed sarcopenia. A conventional logistic regression model including the same covariates produced similar estimates, showed no evidence of lack of fit (*p* = 0.814), and demonstrated acceptable discrimination (AUC 0.784; 95% CI 0.698–0.870).

## 4. Discussion

In this consecutive cohort of patients awaiting primary THA, EWGSOP2-defined sarcopenia affected nearly 15% of the patients and was associated with older age, lower BMI, greater comorbidity burden, reduced functional independence, poorer cognitive status, lower mental health-related quality of life, and worse hip-related function. These findings indicate that sarcopenia identifies a subgroup of patients with a less favorable baseline clinical profile. Although our study was not designed to evaluate postoperative outcomes, these results support the potential value of preoperative sarcopenia assessment for patient characterization and risk stratification. Future prospective studies should determine whether sarcopenia is associated with postoperative recovery, complications, and functional outcomes after THA.

An additional finding of this study is the presence of significant differences across sarcopenia categories defined by the EWGSOP2 criteria. More advanced categories were associated with worsening overall health status, characterized by greater pain, greater comorbidity burden, poorer cognitive status, lower health-related quality of life and worse hip function, although these patterns were not uniformly monotonic across all outcomes. These findings suggest that the sequential EWGSOP2 classification provides clinically relevant stratification beyond a binary diagnosis by identifying patients with less favorable baseline profile before THA. The preservation of the main findings after combining confirmed non-severe and severe sarcopenia also suggests that these associations were not solely dependent on the gait-speed-based severity classification. Whether these baseline differences translate into greater vulnerability or poorer postoperative recovery cannot be determined from the present cross-sectional analysis and should be assessed in longitudinal studies specifically evaluating postoperative outcomes. Previous studies have shown that sarcopenia is associated with poorer functional outcomes, increased complication rates, and greater healthcare costs in patients undergoing total hip arthroplasty [10,12,13,27,28,29]. Together, these findings support the role of sarcopenia as a relevant perioperative risk phenotype and emphasize the need for systematic screening and diagnosis in candidates for THA using standardized diagnostic criteria to improve consistency and comparability across studies [29,30]. In this context, several approaches to case identification and diagnosis have been proposed.

In the present study, sarcopenia was assessed directly using objective measurements of muscle strength instead of relying on SARC-F as a preliminary case-finding tool. Although SARC-F is recommended by the EWGSOP2, its high specificity but limited sensitivity may result in missed cases [31,32,33]. By directly assessing muscle strength, handgrip measurement provides a rapid and practical first-line approach for identifying patients who require further diagnostic assessment.

In our cohort, lower BMI was independently associated with sarcopenia. However, BMI does not distinguish between fat mass, muscle mass, and muscle function and is therefore insufficient as a stand-alone screening measure for sarcopenia in older adults [34]. In contrast, handgrip strength provides a direct assessment of muscle function and has shown stronger and more consistent associations with mobility impairment, disability, and mortality than BMI-based definitions or lean mass alone [35,36]. These findings support the central role of muscle strength assessment within the EWGSOP2 framework and its incorporation into routine sarcopenia screening.

Although muscle and bone deterioration frequently coexist within the muscle–bone unit [37,38], neither Dorr classification nor CCR differed significantly across EWGSOP2 categories after adjustment for multiple comparisons. The association between lower Barthel Index scores and confirmed sarcopenia observed in our cohort suggests that reduced independence in activities of daily living is closely linked to impaired muscle health in arthroplasty candidates. In the multivariable model, each 10-point decrease in Barthel Index was associated with higher odds of confirmed sarcopenia (OR 2.02; 95% CI 1.20–3.60). This finding is consistent with previous evidence reporting lower Barthel Index scores in patients with sarcopenia compared with non-sarcopenic patients undergoing hip or knee replacement [13]. Similarly, poorer Barthel Index scores in sarcopenic THA patients than in non-sarcopenic THA patients before injury, at 3 months, and at 1 year after surgery were found [39]. Taken together, these findings support that sarcopenia is not only a disorder of muscle quantity and strength, but also a clinically relevant phenotype associated with reduced functional reserve before surgery and potentially slower functional recovery thereafter [13,39].

In patients undergoing THA, a minimal EWGSOP2 screening appears feasible and informative: handgrip strength may serve as an initial screening step, followed by DXA-derived SMI assessment when muscle strength is reduced (approximately one quarter of the cohort), and physical performance testing when feasible. This approach allows a more structured characterization of preoperative reserve, rather than providing direct prognostic evidence for postoperative outcomes [40]. However, hip OA may substantially impair gait mechanics (and therefore confound gait-speed-based severity grading). The alternative performance measures recommended by EWGSOP2—most commonly the Short Physical Performance Battery (SPPB), the Timed Up and Go (TUG), or the 400-m walk test [2]—may also be influenced by OA-related pain and load intolerance and should therefore be interpreted as markers of overall functional limitation rather than muscle-specific impairment alone.

Several alternative methods have been proposed for sarcopenia screening and diagnosis. Calf circumference (CC) and BMI-adjusted CC are simple and inexpensive approaches [41,42], although their measurement protocols and cutoff values remain heterogeneous [43,44], and they may also be influenced by muscle disuse associated with advanced age or lower-limb osteoarthritis [45]. Imaging techniques such as CT and MRI provide accurate assessments of muscle quantity and quality but have limited applicability for routine screening because of cost, availability, and, for CT, radiation exposure [2]. Whole-body DXA was selected in the present study as an EWGSOP2-recommended and feasible method for preoperative muscle-mass assessment. Although CT-derived muscle measurements have been associated with postoperative outcomes in THA populations [11,28], the available evidence remains observational and does not support their routine use for sarcopenia screening. Ultrasound has also emerged as a promising alternative because of its accessibility and lack of radiation, although operator expertise is required [46,47]. Finally, the creatinine-to-cystatin C ratio has been proposed as an indirect biomarker of muscle mass, but it should be considered a complementary rather than a standalone diagnostic tool, and no evidence is currently available in THA populations [48,49]. These approaches may complement the assessment of muscle quantity and quality but do not replace the physical-performance component of the EWGSOP2 framework [2]; therefore, gait-based severity and alternative functional tests should be interpreted cautiously in patients with advanced hip osteoarthritis.

Clinically meaningful functional gains with preoperative resistance training have been documented in joint replacement [50], and multimodal prehabilitation—progressive strengthening (including targeted hip-abductor exercises when weakness is evident) combined with nutritional optimization—appears promising for improving preoperative status and supporting recovery [36,40,51]. Sarcopenia represents a modifiable risk factor in this population, and its identification allows targeted interventions to maximize functional recovery. Expert guidance underscores progressive resistance exercise with adequate protein intake as first-line management [52], and integration into preassessment or optimization clinics is feasible and safe [53]. These findings support a risk-stratified approach in which sarcopenia detection may guide individualized prehabilitation and optimization before surgery. Overall, this study provides clinically relevant evidence on muscle health and body composition assessment in THA candidates and may support the integration of sarcopenia screening into preoperative nutritional and functional optimization pathways.

This study has certain limitations that should be acknowledged. The main limitation of this study is its cross-sectional design, which precludes assessment of the prognostic value of sarcopenia. Therefore, although sarcopenia was associated with a more vulnerable preoperative profile, we cannot determine whether these baseline characteristics translate into poorer postoperative recovery, complications, or functional outcomes after THA. Prospective longitudinal studies are needed to address this question. Also, the single-center design and the relatively small number of patients with confirmed sarcopenia may limit generalizability and reduce the precision of some estimates. In addition, the exclusion criteria may have selected a relatively healthier population, potentially underestimating sarcopenia prevalence and limiting generalizability to frailer or more comorbid patients. Refusal to participate and incomplete baseline assessments may have introduced additional selection bias. Therefore, the findings mainly apply to elective THA candidates without severe systemic or functional impairment. As the cohort was not specifically sized for sarcopenia-related analyses, some subgroup comparisons and multivariable results should be interpreted cautiously, given the limited number of confirmed sarcopenia cases. Finally, walking speed, used to define severe sarcopenia, may have been influenced by hip OA-related functional limitation.

Despite these limitations, the study has notable strengths, including its prospective design with consecutive patient enrollment and the use of standardized EWGSOP2 diagnostic criteria, with muscle mass objectively confirmed by DXA, which enhances methodological robustness and comparability with the recent literature. Moreover, the multidimensional assessment of clinical and functional status allowed a comprehensive characterization of the sarcopenic phenotype in patients undergoing THA.

## 5. Conclusions

In patients undergoing THA, sarcopenia affects a relevant proportion of individuals, and greater EWGSOP2 severity is associated with a poorer baseline clinical and functional profile. Older age, lower BMI and lower functional independence are independently associated with sarcopenia. These findings support the value of preoperative EWGSOP2-based sarcopenia assessment to identify THA candidates with greater clinical vulnerability. Whether targeted optimization or prehabilitation strategies improve postoperative outcomes in these patients should be evaluated in prospective studies.

## Figures and Tables

**Table 1:** Baseline characteristics of patients with and without confirmed sarcopenia according to EWGSOP2 criteria. Values are presented as median [IQR] or *n* (%), unless otherwise stated. *p*-values correspond to between-group comparisons.

		Non-Confirmed Sarcopenia (*n* = 173)	Sarcopenia Confirmed (*n* = 30)	*p*-Value
**Anthropometric variables**				
Sex	Male	83 (48.0%)	12 (40.0%)	0.576
	Female	90 (52.0%)	18 (60.0%)	
Age		70 [63–74]	76 [69–79]	0.002
	50–59	24 (13.9%)	0 (0.0%)	
	60–69	61 (35.3%)	8 (26.7%)	
	70–79	77 (44.5%)	15 (50.0%)	
	>80	11 (6.4%)	7 (23.3%)	
BMI		29.4 [26.4–32.9]	27.1 [24.7–28.9]	0.029
	<18.5	0 (0.0%)	0 (0.0%)	
	18.5–24.9	30 (17.3%)	10 (33.3%)	
	25–29.9	66 (38.2%)	15 (50.0%)	
	30–34.9	59 (34.1%)	4 (13.3%)	
	35–39.9	18 (10.4%)	1 (3.3%)	
**Baseline clinical variables**				
VAS (0–10)		7 [7–8]	8 [7–9]	0.223
ASA	I	23 (13.3%)	7 (23.3%)	0.576
	II	115 (66.5%)	19 (63.3%)	
	III	34 (19.7%)	4 (13.3%)	
	IV	1 (0.6%)	0 (0.0%)	
Charlson Comorbidity Index		3 [2–3]	3 [3–4]	0.029
Barthel Index		100 [95–100]	92 [86–100]	0.008
Reisberg GDS		1 [1–1]	1 [1–2]	<0.001
SF-12 PCS		27.40 [23.51–33.12]	27.80 [25.93–32.42]	0.576
SF-12 MCS		47.22 [35.88–55.86]	37.69 [29.33–47.75]	0.029
**Proximal femur radiographic**				
Tönnis	1	5 (2.9%)	1 (3.3%)	0.991
	2	69 (39.9%)	12 (40.0%)	
	3	99 (57.2%)	17 (56.7%)	
Dorr	A	39 (22.5%)	4 (13.3%)	0.576
	B	96 (55.5%)	16 (53.3%)	
	C	38 (22.0%)	10 (33.3%)	
CCR		0.45 [0.40–0.49]	0.47 [0.44–0.51]	0.223
CTI		0.58 [0.53–0.62]	0.56 [0.54–0.61]	0.576
**Laboratory parameters**				
Hemoglobin		140 [133–150]	140 [130–147]	0.576
Albumin		46.0 [44.0–47.0]	46.0 [43.25–48.0]	0.635
**Functional outcomes measures**				
mHHS		48.6 [42.0–55.0]	41.0 [32.5–46.5]	0.029
HOOS-12 (total)	Total	25.0 [18.7–35.4]	25.0 [16.6–33.3]	0.710
HOOS-12	HPain	25.0 [18.8–31.3]	20.8 [12.5–31.3]	0.576
	HADL	25.0 [18.8–37.5]	28.1 [18.8–37.5]	0.941
	HQoL	25.0 [12.5–31.3]	25.0 [12.5–25.0]	0.576
WOMAC	Total	65 [55–75]	68 [60–77]	0.557
	WSymptom	6 [4–7]	6 [5–7]	0.138
	WPain	12 [9–15]	12 [8–14]	0.632
	WFunction	47 [40–54]	48 [44–55]	0.576

Values from χ^2^ test; Fisher’s exact test used when expected counts were <5. Continuous variables compared using Mann–Whitney U test; Reported *p*-values were adjusted for multiple comparisons using the Benjamini–Hochberg FDR procedure. Adjusted *p*-values < 0.05 considered statistically significant.

**Table 2:** Distribution of baseline variables according to EWGSOP2 categories. Values are presented as median [IQR] or *n* (%) unless otherwise stated. Overall *p*-values correspond to between-group comparisons; trend *p*-values correspond to ordered-trend analyses.

		No Sarcopenia (*n* = 146)	Probable Sarcopenia only (*n* = 27)	Confirmed Non-Severe Sarcopenia (*n* = 9)	Severe Sarcopenia (*n* = 21)	Overall *p*-Value	Ordinal Trend *p*-Value
**Anthropometric variables**							
Age		68.5 [62.0–74.0]	74.0 [70.0–78.5]	74.0 [68.0–77.0]	78.0 [73.0–79.0]	<0.001	<0.003
Sex	Males	74 (50.7%)	9 (33.3%)	4 (44.4%)	8 (38.1%)	0.424	-
	Females	72 (49.3%)	18 (66.7%)	5 (55.6%)	13 (61.9%)		
BMI		29.2 [26.3–31.9]	32.7 [28.6–34.4]	24.8 [24.0–27.0]	28.2 [25.5–29.8]	<0.001	0.839
**Baseline clinical variables**							
VAS		7.0 [6.0–8.0]	8.0 [8.0–9.0]	8.0 [7.0–8.0]	8.0 [7.0–9.0]	0.013	<0.003
ASA	I	19 (13.0%)	4 (14.8%)	2 (22.2%)	5 (23.8%)	0.900	-
	II	97 (66.4%)	18 (66.7%)	6 (66.7%)	13 (61.9%)		
	III	29 (19.9%)	5 (18.5%)	1 (11.1%)	3 (14.3%)		
	IV	1 (0.7%)	0 (0.0%)	0 (0.0%)	0 (0.0%)		
Charlson Comorbidity Index		2.5 [2.0–3.0]	3.0 [3.0–4.0]	3.0 [2.0–3.0]	4.0 [3.0–4.0]	<0.001	<0.003
Reisberg Global Deterioration Scale		1 [1–1]	1 [1–2]	1 [1–1]	1 [1–1]	<0.001	<0.003
Barthel		100.0 [95.0–100.0]	90.0 [90.0–95.0]	100.0 [95.0–100.0]	90.0 [85.0–95.0]	<0.001	<0.003
SF-12 PCS		27.35 [23.51–33.33]	28.6 [23.70–30.85]	32.50 [28.90–36.19]	26.53 [23.95–29.00]	0.152	-
SF-12 MCS		48.83 [38.62–56.36]	34.52 [27.66–43.26]	43.46 [34.16–58.40]	32.45 [27.11–44.72]	<0.001	<0.003
**Laboratory parameters**							
Hemoglobin		141.0 [135.0–151.8]	137.0 [126.5–142.0]	133.0 [129.0–149.0]	141.0 [133.0–145.0]	0.062	-
Albumin		46.0 [44.0–48.0]	45.0 [43.0–46.0]	46.0 [46.0–47.0]	45.0 [43.0–48.0]	0.413	-
**Proximal femur radiographic**							
Tönnis	1	5 (3.4%)	0 (0.0%)	0 (0.0%)	1 (4.8%)	0.880	-
	2	57 (39.0%)	12 (44.4%)	5 (55.6%)	7 (33.3%)		
	3	84 (57.5%)	15 (55.6%)	4 (44.4%)	13 (61.9%)		
DORR	A	36 (24.7%)	3 (11.1%)	2 (22.2%)	2 (9.5%)	0.747	-
	B	82 (56.2%)	14 (51.9%)	3 (33.3%)	13 (61.9%)		
	C	28 (19.2%)	10 (37.0%)	4 (44.4%)	6 (28.6%)		
CCR		0.45 [0.40–0.49]	0.46 [0.42–0.52]	0.50 [0.46–0.51]	0.46 [0.43–0.51]	0.162	-
CTI		0.58 [0.54–0.62]	0.55 [0.52–0.63]	0.54 [0.50–0.57]	0.57 [0.55–0.61]	0.499	-
**Functional outcomes measures**							
mHHS		51.0 [41.3–57.0]	38.6 [30.5–47.9]	52.0 [38.0–62.0]	40.0 [32.0–46.0]	<0.001	<0.003
HOOS-12	Total	25.0 [18.7–35.4]	20.8 [12.5–27.1]	35.4 [14.6–41.7]	25.0 [16.6–29.2]	0.113	-
	HPain	25.0 [18.8–31.3]	18.8 [12.5–25.0]	25.0 [12.5–31.3]	25.0 [12.5–31.3]	0.525	-
	HADL	31.3 [18.8–43.8]	25.0 [18.8–31.3]	31.3 [18.8–43.8]	25.0 [18.8–37.5]	0.766	-
	HQoL	25.0 [12.5–31.3]	12.5 [6.3–25.0]	25.0 [12.5–43.8]	25.0 [12.5–25.0]	0.009	0.009
WOMAC	Total	65.0 [53.3–73.8]	72.0 [66.5–77.0]	58.0 [45.0–67.0]	72.0 [62.0–79.0]	0.018	0.034
	WSymptom	5.0 [4.0–7.0]	7.0 [5.0–7.0]	6.0 [4.0–7.0]	6.0 [5.0–7.0]	0.038	0.005
	WPain	12.0 [9.0–15.0]	13.0 [10.5–15.0]	7.0 [5.0–12.0]	13.0 [10.0–15.0]	0.013	0.404
	WFunction	47.0 [38.3–53.0]	51.0 [47.0–57.5]	44.0 [35.0–50.0]	52.0 [44.0–58.0]	0.068	-

Overall between-group differences were assessed using the Kruskal–Wallis test for continuous and ordinal variables and the Fisher–Freeman–Halton exact test with Monte Carlo estimation for categorical variables. Ordered trends were assessed using the Jonckheere–Terpstra test for continuous and ordinal variables. *p*-values from the overall between-group and ordered-trend analyses were adjusted separately for multiple comparisons using the Benjamini–Hochberg FDR procedure. Adjusted *p*-values < 0.05 were considered statistically significant. A hyphen (-) indicates that the ordered-trend result is not reported because the overall between-group comparison was not statistically significant.

## Data Availability

The datasets generated and analyzed during the current study are available from the corresponding author [M.D.C.-R.] upon reasonable request.

## Abbreviations and Units Compliance

ASA	American Society of Anesthesiologists
BMI	Body Mass Index
CC	Calf Circumference
CCR	Canal-to-Calcar Ratio
CT	Computed Tomography
CTI	Cortical Thickness Index
Cr/CysC	Creatinine-to-cystatin C ratio
DXA	Dual-energy X-ray Absorptiometry
FDR	False Discovery Rate
EWGSOP	European Working Group on Sarcopenia in Older People
GDS	Reisberg Global Deterioration Scale
HOOS-12	12-item Hip disability and Osteoarthritis Outcome Score
MCS	Mental Component Summary
mHHS	Modified Harris Hip Score
MRI	Magnetic Resonance Imaging
OA	Osteoarthritis
PCS	Physical Component Summary
PROs	Patient-Reported Outcomes
QoL	Quality of Life
SF-12	12-Item Short Form Survey
SMI	Skeletal Muscle Index
SPPB	Short Physical Performance Battery
RF-CSA	Rectus femoris cross-sectional area
THA	Total Hip Arthroplasty
TUG	Timed Up and Go
VAS	Visual Analog Scale
WOMAC	Western Ontario and McMaster Universities Osteoarthritis Index
